# A theory of public wellbeing

**DOI:** 10.1186/s12889-019-7626-z

**Published:** 2019-10-11

**Authors:** Matthew Fisher

**Affiliations:** Southgate Institute for Health, Society & Equity, Flinders University, GPO Box 2100, Adelaide, 5001 Australia

**Keywords:** Wellbeing, Stress, Social cognition, Public policy, Social determinants of mental health

## Abstract

**Background:**

Wellbeing is seen as a matter of concern for governments and public policy. However, current theories on wellbeing are not well placed to inform this concern, because they fail to take account of and explain evidence on social determinants of mental health.

**Discussion:**

This article proposes a new theory of public wellbeing which does takes account of such evidence, by explaining the role of stress within three basic functions of social cognition. Building on this description, the article then proposes that wellbeing consists in seven basic abilities, which are always developed and exercised (or not) through constant processes of interaction between individual and environment. The article explains why contemporary theories on wellbeing are poorly placed to inform public policy for wellbeing. It also positions the proposed theory in relation to evidence on social determinants of health (SDH) and the associated public policy agenda. It is argued the proposed theory of wellbeing extends on and challenges the SDH policy agenda in relation to the normative target of policy proposals, factors identified as determinants, impacts of determinants on populations, and proposals for political and social change.

**Conclusion:**

Improved theory on public wellbeing can inform policy for wellbeing because it explains the contingent nature of wellbeing within contemporary social environments, and extends understanding of social determinants of wellbeing.

## Background

Wellbeing is now commonly identified as a goal of public policy (e.g. [1–3]). It is argued that governments should use measures of population wellbeing rather than (merely) of economic activity to assess national progress and formulate policy accordingly [4, 5]. If mental ill-health is regarded as a loss of wellbeing, then governments have every reason to take wellbeing seriously as a policy goal, because the human, social and economic costs of mental ill-health are substantial and increasing [6, 7]. So, if governments or international agencies wish to institute policies to promote public wellbeing, what theory of wellbeing might they adopt to define their goal and design their strategies?

The answer is not obvious. Dodge et al. argue that defining wellbeing is difficult and a number of competing approaches are available [8]. The varieties of current theory on wellbeing reflect the long-standing distinction between a hedonic view of wellbeing as a form of good-feeling subjective experience, and a eudemonic view of wellbeing as living in a certain ‘flourishing’ or well-developed manner [9]. Theories may adopt one or other approach, or combine elements of both. We shall consider some examples momentarily. In this article I firstly briefly review some of the main kinds of contemporary theory on wellbeing and suggest limitations of each as a basis for public policy. I then develop and propose a new theory of public wellbeing, which overcomes these limitations, and discuss its implications for public policy to advance wellbeing. Consistent with definitions of public health [10, 11], a theory of *public* wellbeing is defined here as one able to inform public policy or other organised social action to promote individual and population wellbeing equitably, including through action on social determinants of wellbeing.

The starting premise for my initial critique is that a theory of wellbeing able to inform effective public policy for wellbeing must bridge the space between understanding and theorising wellbeing as an attribute of individuals – to do with their subjective experience, psychology and/or behaviour – and contemporary evidence on the impacts of social environments on mental health [12, 13]. (Some theories or definitions of wellbeing incorporate physical health as a key element [14]. I do not address these directly here because, as will become clear, I see physical health as favourable to, but not a defining component of, wellbeing.) After all, there is strong evidence from epidemiological studies indicating that socioeconomic factors causally contribute to incidence of common forms of mental illness in populations [15], resulting in substantial burden of disease and inequities in mental health [6, 16, 17]. There is strong evidence that chronic stress arousal responding to social stimuli is a crucial mechanism mediating the impacts of social environments on mental health [18–21]. If theory on wellbeing doesn’t take account of such evidence or explain to policy makers what can be done to reduce adverse impacts and increase positive impacts of social conditions on mental health, then surely its value as a basis for policy to advance *public* wellbeing is in doubt. Understanding the mediating role of stress is significant because the ability to not only present epidemiological evidence of correlations between social factors and health outcomes but also to explain the causal pathways and mechanisms linking the two is important for uptake of evidence in policy [22, 23].

Let us then consider two main kinds of current wellbeing theory against a criterion of taking account of evidence on social determinants of mental health. Theories of subjective wellbeing (SWB) adopt a hedonic approach and define wellbeing as a subjective experience of happiness or satisfaction with life [24–26]. Much research on SWB focuses on (putatively) measuring wellbeing in populations by asking people to rate their own happiness or satisfaction, and such measures can subsequently be correlated with social factors such as life events or income level [27, 28]. Such research might seem to position individual wellbeing in a social context, but it does not offer any clear theory on *how* social environments causally affect SWB, and does not explain the role of stress arousal in mediating social impacts on mental health. Furthermore, SWB research indicates that most people’s self-assessed SWB is in the positive range most of the time. Thus, SWB theory just seems to miss important parts of the picture concerning social determinants of mental health when, for example, it finds that around 95% of Australians rate their overall satisfaction with life as positive [27], and yet we know that, in any 12 month period, around 20% of the population is subject to a mental health problem [17]. I claim this limits the relevance of SWB theory for public policy.

Other theories on wellbeing adopt a eudemonic approach, and define wellbeing as the individual exercise of psychological or behavioural attributes which (putatively) contribute to living well such as maintaining positive attitudes or having an ability to ‘bounce back’ from challenges [7, 29]. For example, Ryff and Singer’s theory of Psychological Well-Being [30] codifies dimensions of wellbeing such as self-acceptance, autonomy and positive relationships. Again, the specification of such dimensions is then used to design psychological tests to measure them in populations and assess correlations with social factors. The problem again is that such correlations do not explain mechanisms. For example, Ryff and Singer [30] do recognise evidence on population inequalities in mental health and do pay attention to correlations between their measures of wellbeing and biological markers of stress arousal, but do not explain how and why certain social factors affect stress arousal in certain consistent ways, and thereby affect individual and population mental health.

Another major concern about the policy relevance of contemporary eudemonic approaches is that, when applied in practice, they fall prey to the individualist tendencies of biomedical and behavioural approaches to health [31]; and thus conceptualise wellbeing purely in terms of individual behaviour and construct ‘solutions’ accordingly, to fix or improve individuals by training them to adopt positive attitudes or to be more ‘resilient’, rather than to address salient social or economic factors affecting population mental health. These individualised approaches are consistent with the roots of much contemporary wellbeing theory in psychology [13] and also reflect the influence of individualist political values [32]. The burgeoning wellbeing ‘industry’ adds to the dominate theme of individualisation of health and wellbeing in public discourse.

What then of current status of the literature on public health and social determinants of health (SDH) vis-à-vis wellbeing theory and public policy for wellbeing? Clearly that literature has for some time recognised the role of stress as a mediator of social impacts on mental health [20, 33, 34]. However, despite longstanding public health commitments to wellbeing as a goal [35], a clear theory of public wellbeing is missing from the SDH literature. Instead, improvements in measures of population health status used in epidemiological research such as life expectancy, premature mortality, rates of disease or health behaviours serve as the dominant conceptions of ‘health’ in SDH literature and as the presumptive targets for policy proposals [36]. My proposed theory of public wellbeing responds to Richter [37], who calls for an increased focus on theory development in relation to social determinants of health.

Finally, research on SDH has informed a now well-developed agenda for policy action [36, 38]. Thus, if the interest in the end is to inform public policy, one is led to ask; even if SDH literature lacks a theory of wellbeing, would such a theory actually add anything new to the existing SDH policy agenda? I say it would, and the latter part of this article addresses this question.

## A theory of public wellbeing

The preceding critique and the alternative theory of public wellbeing proposed here respond to Huppert et al. [13], who argue that current wellbeing theory fails to adequately integrate perspectives from neurobiology, psychology and social science. The approach to theory development adopted here is integrative, as Huppert et al. suggest. Much of the evidence and some of the conceptual thinking already exists (in various places) to constitute the proposed theory but has not been brought together in a coherent whole. My starting point is to note again the links already made between evidence on social determinants of population mental health and neuroscience research on human stress responses [19, 34], and subsequent recognition of a need to promote mental health at both an individual and social-environmental scale [11]. These links are important, but the integrative perspective that is lacking – the one that can lead on toward a theory of public wellbeing – is to understand the role of stress arousal in social cognition. The following description builds on my previous work, which explains the relevant functions of social cognition in more depth and detail [39].

### Social cognition and stress arousal

Just as circulation of blood is a function of the heart, ‘functions’ of social cognition can be understood as activities carried out by structures of the brain, which subserve our abilities to navigate social environments – the world of other people’s behaviour [40]. There are a number of key brain functions that mediate everyday social cognition and behaviour. Below I describe three of these, which are crucial to understanding the contingent nature of wellbeing in social environments. Brain processes in general are characterised as cognitive or affective in nature [41]. Cognitive processes include perception, memory and associative learning. Affective process are involved in the production of emotions [41, 42] but my focus is on their role in the *motivation* of different kinds of behaviour [41, 43]. Each of the three brain functions described below combines cognitive and affective processes.

#### Goal-directed behaviour

Areas of the prefrontal cortex (PFC) carry out ‘executive’, cognitive functions concerned with the coordination of sequences of behaviour through time, towards a goal [44, 45]. To achieve this, the PFC maintains a behavioural ‘scheme’ combining information from memory with real-time feedback from the body and senses [46]. The maintenance of behaviour towards a desired goal also incorporates an affective, motivational dimension involving increased neural activity stimulated by the neurotransmitter dopamine [47]. At the most basic level, this form of arousal is about positive motivation *toward* an intended goal – about ‘*doing’* and ‘*getting’*. The ability to self-regulate these motivational processes within sustained sequences of purposeful activity is largely nascent at birth and has to be developed through experience within a supportive learning environment.

#### Social monitoring

Cognitive attention on the current environment is particular focused on social stimuli whenever these are present; the physical form, expressions, gestures, voices and behaviour of other people in relation to the self [48]. In social settings the brain is constantly engaged not only in ‘watching’ other people but also in interpreting their behaviour in goal-directed terms (what they are doing), or emotional terms (how they are feeling). It seems our ability to do so comes about in part through rapid cognitive modelling or simulating of other people’s behaviour as if it were our own [48, 49]. Thus, in effect, by such processes we are able to empathetically interpret and anticipate other people’s behaviour by associating the perceived ‘pattern’ of it with aspects of our own experience [50].

The functions of executive control and social monitoring underpinning social behaviour both draw on learned information from previous experiences to represent and interpret the current environment but also use this information to constantly, iteratively *predict* or *anticipate* what is about to happen, with a default assumption that like events will play out in a way similar to past occurrences [20, 50].

#### Evaluation and behaviour change

Although representation of the current environment and production of behaviour relies on information from prior learning, it is equally important to rapidly adjust cognition and behaviour in response to real-time feedback from the environment [46]. Furthermore, within this process of goal-directed behaviour, feedback and behavioural adjustment, there will be occasions when events in the current environment either change unexpectedly – i.e. run counter to current cognitive predictions – or change in ways which (now) predict an *aversive* outcome. In other words, in the latter case, a stimulus is encountered which has been previously associated with a negative, unpleasant outcome. At these times it is important that these changes are recognised and processes rapidly put into action to interrupt and modify behaviour accordingly.

The orbital frontal cortex (OFC) (the underside of the frontal lobe) together with the amygdala are centrally involved in carrying out these functions. In basic terms, the OFC has a particular role to register and record associations between environmental stimuli and states of positive or negative (aversive) affective arousal, and then to monitor real-time events for any such learned associations [51, 52]. In effect the OFC is predictively *evaluating* moment-by-moment events, and can reinforce goal-directed behaviour when success is predicted. However, when circumstances change unexpectedly or an aversive outcome is predicted, the OFC and the amygdala work together to trigger an acute phase of heightened arousal across a number of areas of the body and the brain [53, 54].

One key part of this cascade of heightened arousal is stimulation of the body’s stress system [55]. Human stress systems originate in the brain and trigger arousal of the sympathetic nervous system and hypothalamic-pituitary-adrenal axis, stimulating release of stress hormones including adrenaline and cortisol [19, 20, 56]. Acute stress arousal also stimulates brain structures involved in sensory perception, attention and goal-directed behaviour [41], and is widely understood as an evolved response supporting rapid behavioural adaptation to environmental threats [56–58]. Importantly, this state of general arousal includes increased activity in parts of the brain involved in *interrupting and changing behaviour* [59, 60]. Thus, the overall effect of this cascade of arousal is to ‘switch on’ a set of linked mechanisms able to recognise and respond to unexpected or aversively-associated changes in the (external) environment, and adapt behaviour to these changes.

### Adaptive social behaviour

Together, these functions of goal-directed behaviour, social monitoring and evaluation involving short-term arousal and behaviour change underpin adaptive social behaviour and learning. Although acute stress arousal is associated with overt threats (a large snarling dog, for example) [42], it is are poorly characterised as a ‘fight or flight’ response. Rather, evaluation and stress arousal play an important role in competent adult social cognition, decision making and navigation of the normal range of exigencies in everyday social environments [53, 61]; to sustain goal-directed activity and flexibly respond to behavioural cues from others, in order to avoid anticipated aversive encounters and maintain cooperative relationships.

### Chronic stress arousal and health

As we shall discuss later, the role of acute stress arousal within processes of competent, flexible social cognition and behaviour can contribute to wellbeing. However, it also is a crucial point of linkage with evidence on SDH and, subsequently, an understanding of the contingent nature of wellbeing within contemporary societies. The crucial point is that stress functions renders human beings vulnerable to adverse effects on physical and mental health, especially when social-environmental conditions contribute to a shift from the ‘regular’ role of acute stress in social cognition to chronic stress arousal.

In general terms, the criteria to be met for such a change to occur are, perhaps, deceptively simple. We noted above that part of the evaluation function of social cognition is to register aversive stimuli based on prior learning and trigger an acute phase of heightened arousal supporting modification in behaviour to adapt to (resolve or avoid) the predicted aversive event. The physiological correlate of an adaptive change in behaviour will be a down-regulation of the acute arousal state [57]. However, in this situation of exposure to a stressor stimulus, what is then likely to occur if exposure extends over time, *and* the person in question is not able to identify and implement any behaviour to resolve or avoid the anticipated aversive event? In this situation, basic conditions have been met for a shift from the normal role of acute arousal to one of a chronic state of aversive evaluation and stress arousal [18], simply because, in the situation described, cognitive attention is likely to recurrently re-predict the problem and, with no apparent solution available a ‘collapse’ occurs in predictive expectation of coping. At the neurochemical level, this change is reflected in increased cortisol production [34], and elevated baseline levels of cortisol over time [20].

Adverse effects of chronic stress on individuals’ health are thought to occur in two main ways: chronic stress arousal and associated internal effects in the body and/or brain – over the short or longer term – themselves increase risk of mental and physical ill-health (e.g. [62–64]); and negative psychological states associated with chronic stress contribute to risky health behaviours such as smoking, over-eating or alcohol use as forms of relief seeking [20, 65].

Stress arousal affects hormonal changes, blood pressure, heart rate, and immune system functions. Under conditions of chronic stress, longer terms effects of these changes are thought to increase risk of various forms of physical ill-health, including obesity, metabolic syndrome, insulin resistance, type 2 diabetes, cardiovascular disease and sleep disorders [21, 34]. Chronic stress has been shown to damage chromosomal structure, potentially leading to earlier onset of age-related diseases [64]. Inequality in exposure to stressors is used to explain inequalities in physical health [20, 66].

Stress arousal also involves stimulation of brain structures involved in sensory acuity, attention and goal-directed behaviour [41]. Chronic stress is associated with abnormalities in the morphology or activities of key brain structures involved in stress system arousal [67] and in the activities of the HPA axis; and these abnormalities are associated with common forms of mental illness [21, 52, 56, 68]. Chronic stress has been identified as a likely mediator of increased risk of mental ill-health associated with factors such as: low income, unemployment, job insecurity, low levels of control in the workplace, low control in the home, adverse life events, and insecure or low-standard housing [18].

Cumulative exposure to stressors over the life course correlates positively with worse physical and mental health outcomes, and differences in such exposure contributes to health inequalities between groups according to gender, race and socioeconomic status [66]. Differences between populations in the direct or indirect effects of chronic stress on health are invoked to help explain associations between socioeconomic status (SES) and health, and other health inequalities within or between countries [69, 70]. Finally, unresolved chronic stress is not only harmful to health but constitutes a form of psychological suffering.

### Issues for wellbeing

Ryan et al. argue that human beings are intrinsically motivated to be active, to do things, to be inquisitive [71], and this I suggest is the same motivation that drives goal-directed behaviour as part of social cognition. It is important therefore that people learn to self-regulate intrinsic motivation, in order to exercise some autonomy and engage in constructive behaviour to achieve valued goals. Doing so feel good. Conversely, experiencing a lack of control in goal-directed tasks increases stress demand [55] and can have adverse effects on mental health if extended over time [72]. Thus, I propose that self-controlled, goal-directed behaviour is part of wellbeing, as others have concluded [7, 29]. However, if that is accepted then a number of further considerations come into focus to refine and qualify this claim – all of which have to do with how abilities of constructive, goal-directed activity are attained and exercised (or not) within a social milieu.

As discussed earlier, much goal-directed activity occurs within social environments and is shaped by perceptions and learned expectations about other people’s behaviour and attitudes towards the self. Where the behaviour or attitudes of others are perceived (possibly sub-consciously) and evaluated as a form of challenge, a potential threat to control or social standing, then they become a stressor [20, 34, 55]. A key question then concerns the kinds of internal resources or external opportunities the person has available, or the kinds of learned expectancies or behaviours they bring to the situation. Part of the optimal self-regulation of goal-directed behaviour in complex social environments is about flexible adaptation to such challenges.

Considering these questions takes us to matters of child development and learning. When a child at around 2 to 4 years old faces an imposed (non-violent) constraint on an ‘anti-social’ behaviour from a parent or carer, that will (no doubt) be experienced as an acute stressor, and the question then is about what the parent does to guide a change in behaviour. Through repetitions of such experience, social cues of disapproval can give rise to short bursts of stress arousal, leading in turn to modified behaviour that is effective in ‘solving the problem’. This is part of what it means to self-regulate motivated, *social* behaviour. The internal resources gathered from such experiences support a child’s capacities at 5 or 7, when faced with a social challenge or expectation (say, to complete a task at school), to generate a state of positive expectancy in response [20]. On the other hand, children subject to chaotic, traumatic or unstimulating environments are likely to develop and repeat other kinds of motivated responses to such everyday social challenges, perhaps characterised by aggression or withdrawal. Children exposed to such environments tend to demonstrate increased sensitivity to stressors in later life, and increased risk of ill-health [34].

A second important point is that time-extended goal-directed activity within complex social environments (outside the typically small circle of close, most-trusted others) just *is* psychologically and physically demanding, because it is likely to involve repeated episodes of acute stress arousal [73]. However, the extent of that demand (and the question of whether it ‘converts’ to chronic stress) will depend on the nature of the task and situation, as well as the resources the individual brings to it. Research on work environments, for example, indicates that work in which an individual can exercise skills and a measure of control is less stress-demanding than work that is tightly prescribed, closely monitored, mundane or repetitive [63, 74]. Control over work or life circumstances is now seen as an important psychosocial determinant of health in its own right, on the premise that it moderates or exacerbates stress demand [75]. Thus, not all goal-directed activity is equal when it comes to benefits for health and wellbeing. A focus on individual coping or resilience as putative features of wellbeing (as per current theory) overlooks the possibility that coping under conditions of constant demand – even if better than not coping – will still result in a high stress load.

A third point is that, within complex social environments, an individual’s belief that others are perceiving her or him in a negative light, sometimes referred to as ‘negative social evaluation’ can operate as a significant stressor [55, 70], again depending on the situation, the resources a person brings to it, and perhaps to heightened sensitivity due to earlier life experiences [34]. To some extent this is part of the normal variety of human relations and what matters is having some strategies and resources to cope. If the situation persists and is felt by the person subject to it to be outside of their control, then it is likely to cause chronic stress arousal. Our sensitivity and aversive response to negative social evaluation also renders us vulnerable to manipulation, because a threat of negative social evaluation and isolation from a group can be used to enforce conformity.

The three points made above make clear the contingent nature of positively motivated, flexible goal-directed activity conducted within complex social settings as an aspect of wellbeing. Having made those points we may now shift the discussion to another track, by recognising that there is a family of psychological states and activities that sit ‘outside’ this kind of socially-engaged, goal-directed activity which, even in the best of circumstances, will involve the demand of sustained arousal and stress. These ‘other’ kinds of activities share the characteristic of avoiding this demand, and open up other dimensions of wellbeing. Again, there are three points.

First, it is crucial to observe that positively motivated goal-directed activity can and does occur in ways that are not engaged with complex social environments and thus do not involve repetitive (stressful) demands for attention on other people’s behaviour. Consider the difference between travelling to a city to do paid work, interacting with others, meeting deadlines and so on, and building a model ship or working in the garden at home. The salient point about the latter kinds of activity is that they are largely self-controlled and offers the rewards of applying skills to attain a goal, but are not exposed to the stress-inducing demands of navigating complex social environments. Thus, there are two distinct kinds of goal-directed activity related to wellbeing – a point implied but not made clear in other theory [29].

Second, while ideas of individual goal-directed action as a feature of wellbeing may seem (or be used) to justify the individualist values of modern, market societies [32], in fact there is a raft of evidence to suggests that the experience of positive, reciprocal social relations and activity contributing to the wellbeing of others are important contributors to health and happiness (e.g. [76, 77]). Such evidence is consistent with my description above of the social monitoring function of social cognition.

Third, a significant body of evidence indicates potential benefits for improving wellbeing and reducing symptoms of mental ill-health from activities that shift attention away from predictive, future-focused task orientation and onto more present-time experience of the senses, the body, aesthetic experience, and/or the natural environment [78, 79]. This present-focused state of mind can be achieved through contact with nature, meditation, making art, play, eating, listening to music, and engaging in physical exercise. Indigenous peoples’ cultures commonly place high value on relatedness with the natural world, and this is highly relevant to understanding wellbeing [80].

In modern social environments where goal-directed, socially-engaged activity occupies much of people’s time, a cognitive focus on ‘the task’ can often demand attention and elicit stress arousal well beyond designated work hours, even interfering with sleep. The kinds of present-focused activities described above are important opportunities to ‘down-regulate’ this kind of habituated arousal. Again, it is essential to recognise that the ability to self-regulate cognitive attention, behaviour and affective arousal in these ‘other’ ways (as described) is in part a learned skill – a quite different form of using one’s time constructively. Many people, one suspects, do not have such skills or do not recognise their value. In fact, the more common response is to attempt to self-manage the chronic, felt demand and stress of modern life in more diversionary ways such as watching television, gambling or using drugs and alcohol [65], which are less effective and may have negative impacts on health. However, access to the kinds of stress-reducing activities described may also be determined by social conditions such as income, social relatedness (or isolation), or access to natural environments.

Furthermore, looking beyond this rather limited picture of occasional opportunities for recovery from the demands of modern life, I suggest that optimal conditions for wellbeing will involve a far more even balance between periods of socially engaged goal-directed activity and periods of time dedicated to self-controlled, goal-directed action, sociality and the well-being of others, and the present-focused activities of sensory and aesthetic experience and contact with nature.

### Defining wellbeing

Based on the above discussion, I define individual, adult wellbeing as having and exercising seven basic *abilities*. These are abilities to:

1. Engage in sustained, constructive, self-controlled goal-directed activity within complex social environments, in ways that exercise skills, achieve valued or meaningful outcomes, and avoid chronic stress
2. Respond constructively to social challenges and rapidly adjust behaviour in response to social cues and norms
3. Engage in self-controlled, creative, goal-directed activity ‘outside’ constraints of social demands and expectations
4. Engage in and enjoy positive, reciprocal social relationships and contribute to the wellbeing of others
5. Engage in present-focused activities of a sensory, meditative, creative, playful or aesthetic nature including regular contact with nature.
6. Achieve a balance between the demands of socially-engaged, goal-directed activity (points 1 and 2) and other kinds of activity (points 3–5)
7. Understand the nature of wellbeing and the social and environmental conditions required to attain it, and work to ensure these are available to the self and others

From a life course perspective, wellbeing involves children and adolescents gradually developing the cognitive resources and associated behavioural skills to *self-regulate* the abilities of wellbeing, through (guided) opportunities to *exercise* them in simple forms. Furthermore, the points above are not intended to be exhaustive. In particular socio-cultural environments, other general living skills such as literacy or cooking skills will assist the exercise of wellbeing abilities. Such learnings can form part of the self-regulation of behaviour that is part of well-being.

Clearly, this is a eudemonic view of wellbeing, but it is important to say that developing and fulsomely exercising the abilities described above will likely result in regular subjective states that individuals might variously describe as happiness, calm or satisfaction [13]. Without this association with experience, the abilities described would be of lesser value. However, the merit of a eudemonic view is that it take us beyond subject experience and opens the way to recognise that wellbeing (as a way of living) is always realised or not, through processes of *interaction* between the individual and his or her socio-cultural milieu.

To expand on this point, at a first level of analysis, we can say that, in any particular situation, the actual exercise of wellbeing abilities will always occur (or not) as an *interaction* between the internal resources and dispositions an individual brings to the situation, and the objective nature of the situation itself [20, 81]. Part of what an individual has to call on in a demanding situation may also be to do with their personal, social and economic circumstances. At a second level of analysis, proceeding from the developmental aspects of wellbeing, we can say that the resources and dispositions an individual brings to a situation – at any point in time – will themselves be largely determined by a series of prior interactions between the internal and external milieu, going back to in-utero development. This recognition of the fundamentally *transactional* nature of wellbeing, and the permeability of the individual to its environment, is the key to avoiding the mistake of individualisation and instead combining an individual and a population-based view of wellbeing.

Thus, we may theorise that wellbeing in populations and inequalities in wellbeing between groups defined by SES or other attributes will be shaped by the social conditions they are exposed to, as these support/do not support: a) the development of cognitive, affective and behavioural resources for self-regulation of wellbeing abilities; *and* b) the opportunity to exercise wellbeing abilities in particular situations. Inequalities in conditions supportive or not supportive of wellbeing are likely to be shaped by the structural socioeconomic inequalities typical of modern societies with market economies [15].

The penultimate point is to emphasise that the neuropsychological functions of evaluation constitute an act of (conscious or subconscious) *interpretation* of the social environment. This means that *beliefs about the world* as it affects the self, enter the equation of wellbeing and stress arousal [82]. For example, if I lied, and told you that senior managers at your workplace were reviewing your performance because of some concerns raised, this belief alone would very likely give rise to elevated stress arousal, even though the social-evaluative ‘threat’ in this case was entirely in your head, and actually fictitious. If your housing or family situation were financially tenuous, the stress demand of this belief could be exacerbated. As we shall discuss below, this potential role of beliefs (whether accurate or not) about the world, and about how other people perceive you, as a mediator of goal-directed behaviour and stress arousal has significance that goes well beyond the situations of individuals.

Finally, some comments on point seven concerning a working *understanding* of the nature of personal and public wellbeing. This point is somewhat distinct from the others because – I think – it is possible to exercise abilities one to six, and thereby enjoy wellbeing without necessarily explicitly understanding the nature or terms of that experience. One might even suppose that such understanding, in a world antagonistic to wellbeing in many respects, might in fact detract from the experience. Nevertheless, understanding warrants inclusion in the scheme as a ‘meta-ability’ for wellbeing. It provides for a deliberate practice of wellbeing, with awareness of the potential impacts of social conditions on the self. It allows for thoughtful action in social relationships or in the broader spheres of public policy or cultural beliefs and practices.

## Implications for wellbeing theory and SDH policy agenda

### Implications for wellbeing theory

The proposed theory is better placed than other contemporary theories to inform public policy and social action to promote wellbeing, because it can explain the nature and dynamics of wellbeing in populations and how these are shaped by social conditions. Other theories discussed earlier start from the position of a psychologist and posit views of wellbeing as consisting in certain individual psychological experiences, attributes or behaviours, and then consider how these may be related to social conditions. The theory proposed here starts from evidence in neuroscience on the brain functions underpinning social cognition. These functions are understood from the outset to be constantly interacting with – drawing information from and responding to – the external environment. The role of stress arousal is built into the theory as an element of social cognition and thus the theory is able to explain how and why – in what kinds of conditions – humans are vulnerable to chronic stress and its adverse effects on subjective experience (distress), social behaviour and mental health. The proposed theory is supported by convergent evidence from different disciplines. Evidence from neuroscience studies on the nature and functions of stress arousal in animals are consistent with (and help to explain) evidence from psychological studies on stress and from epidemiological studies on associations between exposure to certain kinds of social conditions and effects on mental health [39]. However, while the points of connection between research on stress and on social determinants of mental health have been recognised for some time in public health literature [19, 33, 34], they have not been integrated into a theory of public wellbeing. In these ways the proposed theory meets Huppert et al.’s call for theory on wellbeing integrating perspectives from neurobiology, psychology and social science.

Thus, although the proposed theory speaks to the nature of wellbeing in the individual, wellbeing is not defined as consisting in intrinsically individual psychological experiences, attributes or behaviours. Rather, wellbeing is defined in contingent terms as the having and exercising of certain abilities, which are *always* developed and exercised (or not) through constant processes of interaction between individual and environment.

When the contingent nature of wellbeing abilities are understood – including the mechanism of stress arousal, its role in social cognition and the kinds of conditions that cause chronic stress – then one is in a position to understand how social environments impacts on wellbeing in individuals and in populations and take action accordingly. This is about understanding and cultivating conditions that support wellbeing but it is also about understanding what is at stake for us if the social conditions we create together fail to cultivate wellbeing: not just mental ill health but also psychological suffering, and increased levels of disturbed, divisive or destructive social behaviour. It is in these ways that the proposed theory is suitable to inform public policy and social action to promote wellbeing – to be a theory of *public* wellbeing.

### Implications for the SDH policy agenda

The proposed theory of wellbeing does not fundamentally disagree with the SDH policy agenda, which for current purposes I will take to be exemplified by the final report of the WHO Commission of Social Determinants of Health [36] and similar publications that review SDH and propose a ‘package’ of policy measures accordingly [38]. However, perspectives on policy and social change arising from proposed theory of wellbeing overlap with but also challenge and extend on the SDH policy agenda in a number of ways. Here it is only possible to summarise some key points, and each of these will require further exploration elsewhere.

Firstly, the goals of public health have long been couched as a state of wellbeing that goes beyond (merely) the absence of illness [35]. However, despite this, because the SDH policy agenda is largely built on epidemiological research it tends to position improvements in measures of health used in such research – life expectancy, premature mortality, rates of disease, disease burden, disease risk and so on – as the intended goals of its proposals. Reducing socially determined inequalities in these measures between population groups is often a main focus. Thus, although the underpinning values of the agenda are expressed as ‘health’ and ‘health equity’, in operational terms health *is* largely defined as the absence of illness and there is no substantive, coherent conception of wellbeing present as an intended target of policy. While reductions in common forms of physical or mental illness are important policy goals in their own right and being subject to such ill-health conditions is likely to undermine wellbeing, the (mere) absence of these conditions does not *constitute* wellbeing as I’ve defined it. Thus, identifying wellbeing as such as a normative goal of public policy – supported by an applicable theory – does extend on the goals of the contemporary SDH policy agenda. Furthermore, a focus on wellbeing can extend one’s policy agenda into a space where it can question broader political values – beyond the value assigned to ‘health’ as commonly understood – because claims about wellbeing (or welfare), and the capacity to ‘deliver the goods’ for human welfare, are central to the moral legitimation of contemporary political systems [39].

The proposed theory of wellbeing also challenges the SDH policy agenda because it extends on the range of factors identified as determinants, which serve as the substantive targets for policy activity. The SDH policy agenda proposes that governments act on factors such as education, income, employment, housing, gender and racial discrimination and access to healthcare in order to improve health or reduce health inequities [36]. Such actions are relevant for wellbeing, because many of these factors are known to affect risk of mental ill health. However, the proposed theory and its analysis of the functions of social cognition draws attention to other factors and effects on populations that are barely recognised in the SDH policy agenda. Here I will focus on two issues; the role of beliefs and time-extended stress demands of modern environments. As mentioned above, the taking on of particular beliefs about the world can significantly affect stress arousal, because much stress arousal in adults starts with processes of cognitive interpretation and evaluation of the world as it affects the self. Thus, the public promulgation of certain kinds of ideas can exploit human vulnerabilities to stress arousal, when they are taken on as beliefs about threats. The representation of some ‘other’ group as a threat to a person’s perceived identity group (to ‘us’) can be used as a tool to manipulate political support precisely because it taps into this vulnerability. If citizens are already under stress due to other demands, then their vulnerability to such exploitation is likely to be higher. Such manipulation is well-recognised as a sociological problem; the proposed theory can explain *why it works*. Wellbeing as a public goal thus brings the ideational environment of public discourse within the scope of issues to which public policy should pay attention. Similarly, awareness of climate change as a real existential threat has the potential to act as a chronic stressor and contribute to increased psychological distress and metal ill-health on a global scale – especially if there is no sense of being able to *act* meaningfully to address the threat.

The proposed theory also explains that undertaking goal-directed, socially engaged behaviour is intrinsically stress-demanding and will be more so if perceptions of control are limited. Thus the theory specifies and explains a ubiquitous feature of modern, competitive, hierarchical societies as a determinant of wellbeing; that is, an increased and time-extended demand for cognitive attention on goal-directed behaviour within complex social environments, occurring in a socioeconomic climate where, for many, employment conditions are relatively insecure. Part of this is about the demands of extended hours ‘out there’ in the real world, getting to and from work, meeting demands and deadlines and so on. An important additional factor is the role of digital media in all forms. Social cognition, being highly sensitive to social stimuli, will generally not neatly discriminate between direct human interaction and virtual social environments created though digital media. These media are able to bring stress-inducing, attention grabbing stimuli into our awareness 24 hours a day, seven days a week; whether these are to do with our employment or social networks, or violent and unsettling events from around the world, or even fictionalised violence. This is quite new for humanity and we have barely come to terms with its implications for wellbeing. It is not a well-recognised issue in the SDH policy agenda.

The theory of public wellbeing also challenges and extends perspectives on the impacts of determinants. It indicates that the ability to self-regulate social behaviour in pro-social ways will be affected by present time events in the surrounding social milieu and by a person’s history of interactions with her or his social environment. Thus failures in the development or exercise of wellbeing abilities is likely to adversely affect social behaviour. For example, based on the proposed theory I would suggest that the experience of chronic stress may alter a person’s perceptions of other people with whom they are interacting; to see them as *sources of demand* even if they have little to do with the actual stressors affecting that person’s life. These perceptions may result in a loss of empathy [83], or stimulate aggressive or defensive withdrawal behaviours. I noted earlier evidence that chronic stress may also stimulate or increase relief-seeking through behaviours such as smoking or use of alcohol [65]. When the current SDH policy agenda concerns itself with psychology it tends to focus on that which epidemiology measures – the presence or absence of common forms of mental ill-health [16]. The proposed theory suggests that adverse impacts of chronic stress may in fact have effects on populations and raise policy concerns that go far wider than high rates of diagnosable mental health problems, concerning as these may be; including issues such as family and social violence, various forms of addictive behaviour, and psychological suffering.

The focus of the SDH policy agenda on measures of disease makes it relatively easy for policy makers to convert the concerns raised into reasons to deliver healthcare or social policy interventions [84]. To bring policy attention onto wellbeing – informed by suitable theory – on the other hand, would place an onus on policy makers to shift focus away from ill-health and toward discerning and cultivating the conditions required for the development and exercise of wellbeing. In this sense, the proposed theory also responds to Antonovsky’s call for a genuinely salutogenic approach to health promotion, moving beyond the narrow policy obsession with health behaviours [85]. I say it is through an understanding of the transactional nature of wellbeing across the life course, and the mechanisms that mediate social impacts on wellbeing, that the essential conditions required for the development and exercise of wellbeing can be discerned and become the subject of policy attention. Without this understanding the notion of governments measuring wellbeing as a means to stimulate relevant action is fundamentally flawed.

Finally, specifying in detail the social conditions required for the development and exercise of wellbeing abilities will be essential to augment the proposed theory but is a task for another time. However, the theory already points out the direction of travel. Clearly conditions of early life from conception to age 10 or so are crucial for development of basic resources for self-regulation of behaviour, navigating social relationships in pro-social ways, and adaptively coping with – or being resilient to – social stressors [86]. It follows, therefore, that social, economic or cultural factors affecting parents’ mental health and wellbeing, and sensitivity to stressors, must also be recognised as matters of fundamental concern; for their own sake of course, but also because they may affect people’s behaviours as parents, with adverse consequences for their children’s exposure to stressors and prospects for wellbeing in the longer term [11].

In relation to adult life, we clearly need social and economic conditions that value and cultivate the mix of abilities indicated in the proposed theory more effectively. It is also important to recognise that the abilities of wellbeing are in significant part developed and exercised in the medium of human relationships as these play out in relatively intimate or localised spaces of family, friends, workplace or community. In this sense, human communities should be seen as a fundamental foundation for social wellbeing. While the provision of public services in areas such as education and healthcare is essential, the proposed theory also suggests a need for people to be, not just passive recipients of services, but active, knowledgeable participants in a social project of cultivating wellbeing. Wellbeing abilities are developed and maintained by being *exercised*.

## Conclusion

Current wellbeing theory is not well-placed to inform public policy for wellbeing. The proposed theory of public wellbeing can inform policy for wellbeing because it explains the contingent nature of wellbeing within contemporary social environments. When this theory is applied to examine current conditions then issues of heart disease or cancer must be re-assessed as second-order issues beside the potential cumulative impacts of social, economic, environmental and cultural stressors on human psychology, social behaviour and mental health. However, at the same time, understanding of the essential conditions required for wellbeing has the potential to inform political and social change toward a different future.

## Data Availability

Not applicable.

## Abbreviations

HPA axis: Hypothalamic-pituitary-adrenal axis
OFC: Orbitofrontal cortex
PFC: Prefrontal cortex
SDH: Social determinants of health
SWB: Subjective wellbeing
